# A Superhydrophobic Moso Bamboo Cellulose Nano-Fibril Film Modified by Dopamine Hydrochloride

**DOI:** 10.3389/fbioe.2021.756839

**Published:** 2021-10-20

**Authors:** Yan Wu, Wanying Zhao, Xinyu Wu, Jian Gan, Haiqiao Zhang, Yijing Cai

**Affiliations:** ^1^ College of Furnishings and Industrial Design, Nanjing Forestry University, Nanjing, China; ^2^ Co-Innovation Center of Efficient Processing and Utilization of Forest Resources, Nanjing Forestry University, Nanjing, China

**Keywords:** bamboo fiber, cellulose nano-fibril, film, contact angle, superhydrophobicity

## Abstract

The moso bamboo fiber powder was used as raw material to prepare cellulose nano-fibril films, 5% of polyvinyl alcohol solution was used as a structural reinforcement agent, dopamine hydrochloride (DA) was used as a surface adhesive, and hexadecyl trimethoxy silane was used as a surface modifier. The superhydrophobic films were prepared by vacuum filtration and impregnation. The results showed that the water contact angle on the surface of the film could reach 156°. The microstructure and chemical composition of the film surface was further studied by scanning electron microscopy (SEM), Fourier transforms infrared spectroscopy (FTIR), and roughness measurement The scanning electron microscopy images showed that the nanofibers on the surface of Cellulose nanofibers film were arranged and randomly distributed, thus forming a dense network interwoven structure. In PDA hydrophobic modification solution, an Hexadecyltrimethoxysilane was hydrolyzed to a hexadecyl silanol to obtain the polar terminal hydroxyl of Hexadecyl silanol molecule. The -OCH3 terminal group of HDTMS reacted with hydroxyl/H_2_O to form a silanol (Si-OH) bond and further condensed to form a Si-O-Si network. In addition, due to the hydrophilicity of the surface of the nano cellulose film, a large amount of—OH was adsorbed on the surface of the nano cellulose film, resulted in the chemical connection between cetyl groups, thus realized the grafting of cetyl long-chain alkyl groups onto the fibers of the nano cellulose film.The film showed good self-cleaning and waterproof properties, which can be widely used in wet environment packaging and building.

## Introduction

In recent years, cellulose from agricultural waste, industrial processing residues and energy crops has been widely used in construction, packaging, pulp production (Rangaraj and Venkatachalam, 2017), textile manufacturing and wastewater treatment, etc. The fibers in natural cellulose were hydrophilic and prone to erosion in wet environments. Therefore, improving the hydrophobic properties of cellulose surface will provide more possibilities for packaging and construction materials (Liu et al., 2019a).

Now, most of the polymer materials in the production process are synthesized from petroleum and coal products, including film, tire, silk, and so on. The polymer produced from petroleum has a great impact on environmental pollution since it cannot be biodegraded after being discarded (Lu et al., 2021). As renewable, biodegradable, low-density, non-toxic, green resources, biomass nanofiber materials with excellent mechanical properties have been widely concerned by scholars (Wu et al., 2019a). In recent years, researchers began to use bamboo processing residues as raw materials to prepare nano cellulose (Tsubota et al., 2018). Bamboo has a short growth cycle and fast growth rate, so it is a very potential biomass material (Wang et al., 2019a). Han et al. prepared Bamboo powder cellulose nanofibers (BCNF) aqueous suspension by extraction, bleaching, alkali treatment, and ultrasonic treatment of bamboo powder. The water in the sol was removed by vacuum filtration of Cellulose nanofibers (CNF) aqueous suspension, which was transformed into CNF film after drying treatment. CNF has a large specific surface area and high aspect ratio. Under the action of hydrogen bonds, CNF films with good mechanical properties and light transmittance are formed. CNF composite film can be prepared by mixing with polyvinyl alcohol. Polyvinyl alcohol is a kind of polyhydroxy water-soluble polymer (Wu et al., 2019b). The film material made of polyvinyl alcohol has good flexibility, transparency, and wear resistance, and can be biodegradable under certain conditions. The results showed that the CNF film prepared by bamboo powder combined with polyvinyl alcohol is of great significance to protect the environment and reduce secondary pollution.

When the surface contact angle of water is greater than 150, the object surface shows superhydrophobic characteristics. Many plants and animals in nature have superhydrophobic functions, such as lotus leaves, butterfly wings, clover. By observing the microstructure of the plant surface, the experimenter found that the existence of a wax layer on the plant surface is the main reason for achieving superhydrophobic and self-cleaning properties (Bhushan et al., 2009). The three-dimensional structure is constructed on the surface of the material, so that the surface has the property of low surface energy. At the same time, the hydrophobic waxy material is introduced into the surface of the material, which can effectively prevent the penetration of water droplets, so as to show the hydrophobic performance.Inspired by the superwater of natural plants and animals, the bionic modification of superwater phosphorus surface is created on the film surface. There are strong hydrophilicgroups on the surface of CNF film material, so it is easy to absorb water. To improve the application of film materials in a humid environment, hydrophobic groups are usually introduced to the surface of film materials by hydroxysilanization to achieve a hydrophobic effect (Barthlott and Neinhuis, 1997).

The main methods for preparing superhydrophobic film surface are impregnation method, chemical vapor deposition, spraying method (Wang et al., 2016), sol-gel method, etching method, electrospinning.Two principles are mainly used: one is to reduce the surface energy of the material; the other is to construct a rough structure on the surface of the material first, and then modify it with low surface energy material. Wang et al. used the electrospinning process to mix CNC into PVDF to produce hydrophobic and oleophilic nano-cellulose (Wang et al., 2019b). Zhang et al. modified the surface of stainless steel with cetyltrimethoxy silane to make it have superhydrophobic properties and corrosion resistance (Zhang et al., 2020). Wang et al. prepared superhydrophobic films by configuring cetyltrimethoxy silane to protect the surface of aluminum alloy (Rahimipour et al., 2020). The results show that the long-chain organosilane in cetyltrimethoxysilane can reduce the surface energy and enhance the hydrophobicity of the film surface. Therefore, the introduction of cetyltrimethoxysilane into the preparation of nano cellulose superhydrophobic film has a wide application prospect. In addition, the CNF superhydrophobic film treatment method has high efficiency, low pollution, low energy consumption, and good self-cleaning effect.

Inspired by the protein mucus secreted by marine mussels, modern researchers have found that the catechol group in PDA can interact with chemical reactions and substrate materials through various interactions. Dopamine hydrochloride contains catechol and amino molecules, which self polymerize to form polydopamine in a mild reaction environment with oxidant and weak base (El Yakhlifi and Ball, 2020). Polydopamine has strong adhesion, by soaking the solution and then combining it with various functional substances to adhere to various substrate materials to achieve the purpose of a secondary reaction, the surface rough structure or low surface energy structure can be constructed, and the surface hydrophobic modification of the substrate materials can be realized (Cheng et al., 1021). In addition, it is found that the H+ in the catechol group is easily lost in the strong alkali condition. PDA has a rough alkali etching structure due to the destruction and decomposition of hydrogen bonds in the molecule. This method can also realize the hydrophobic treatment of the substrate. The wood coated with polydopamine was stirred and dried in a strong alkaline solution, and then treated with Octadecyltrichlorosilane (OTS) for low surface energy to obtain superhydrophobic wood. With biodegradability, biocompatibility, and unique physical and chemical properties, PDA is widely used in biomedicine, environment, energy, and other fields. Wang et al. used dip-coating technology to soak the fabric in the solution composed of dopamine/Hexadecyltrimethoxysilane (HDTMS) to obtain fluoride-free and durable superhydrophobic fabric (Wang et al., 2017). PDA can be easily synthesized in alkaline medium by oxidative polymerization of dopamine, and the rich functional groups in PDA, such as catechol and amine, made it an attractive polymer that can be used to modify the film surface. Catechol in the polydopamine coating will react with hydroxyl groups in cellulose to give cellulose strong binding force and improved the mechanical strength of cellulose film. Catechol tended to react with hydroxyl groups, resulted in dehydration and the formation of charge transfer complexes, so that the surface of CNF/PVA film was coated with uniform dopamine solution. PDA can scavenge free radicals and enhance the chemical stability of polymers. Dopamine as a medium can improve the adhesion between the long-chain alkyl in cetyl and CNF substrate, so as to ensure the excellent stability of CNF/PVA/PDA composite film. The results showed that PDA enhanced the adhesion of hydrophobic functional materials on a solid surface.As a natural cellulose material, bamboo powder had green environmental protection, degradability and biocompatibility. It can be widely used in packaging and pulp production. In this paper, bamboo powder was used as the substrate, and the CNF composite film was chemically modified with dopamine to prepare the superhydrophobic film. It was used as a functional material to retain the natural characteristics of bamboo powder. This study theoretically discussed the friendly and environmental protection type. It can be widely used in flexible electronic products (Shan et al., 2020), food packaging (Chen et al., 2020).

## Experiment

### Materials

The bamboo powder was taken from Yihua Lifestyle Technology Co., Ltd., Shantou, China, which was the processing residue of the production workshop. Most of the shredded material was fibrous, ventilated sodium hypochlorite (NaClO2) and DA were provided by Shanghai Aladdin Biochemical Technology Co., Ltd., ShangHai, China. Acetic acid (CH_3_COOH, 99.5%), ethanol absolute (99.7%) and sodium hydroxide (NaOH) were all supplied by Nanjing Chemical Reagent Co., Ltd., NanJing, China. Polyvinyl alcohol 124 (PVA) was obtained from Sinopharm Chemical Reagent Co., Ltd., ShangHai, China. Hexadecyltrimethoxysilane (HDTMS) was provided by Shanghai Eon Chemical Technology Co., Ltd., ShangHai, China.

NaOH solution was purchased from Dongguan Jinzhida Technology Co., Ltd., Dongguan, China.

### Experimental Methods

#### 2.2.1. Preparation of CNF

The bamboo powder was sieved through 60 mesh sieve and dried in an oven (Shanghai Xinmiao Medical Instrument Co., Ltd., Shanghai, China) at 105°C for 4 h; 5 wt% sodium chlorite was poured into bamboo powder, put into the water bath (Changsha Tuan Electromechanical Automobile Equipment Co. Ltd.) and heated at 75°C for 2 h, After that, the waste liquid was poured out, and 5 wt% sodium chlorite solution was prepared, which was poured into a beaker with bamboo powder and heated in a water bath for 1 h; 2 wt% NaOH water bath was used for 2 h; Cycled the above two steps once. Finally, the pH value was adjusted to seven by acetic acid. After washing with distilled water, the suspension was white and sealed with plastic film.

#### 2.2.2. Preparation of CNF/PVA

Firstly, 20 g of 10% CNF suspension was added with 60 ml of water and treated by ultrasonic crusher (misonix, Inc., New York, United States) for 30 min; The obtained solution was filtered by a vacuum pumping device to obtain a layer of nano-cellulose wet film. The wet film was dried at room temperature for 2 h, and the wet film was removed from the filter paper. The wet film was sandwiched between two layers of paper and let stand for 1 h. 5% PVA solution was prepared and the wet film was.

Impregnated in PVA solution for 24 h. After that, the wet film was taken out and dried in an oven with a temperature of 50°C for 30 min.

#### 2.2.3. Preparation of CNF/PVA/PDA

**FIGURE 1:**
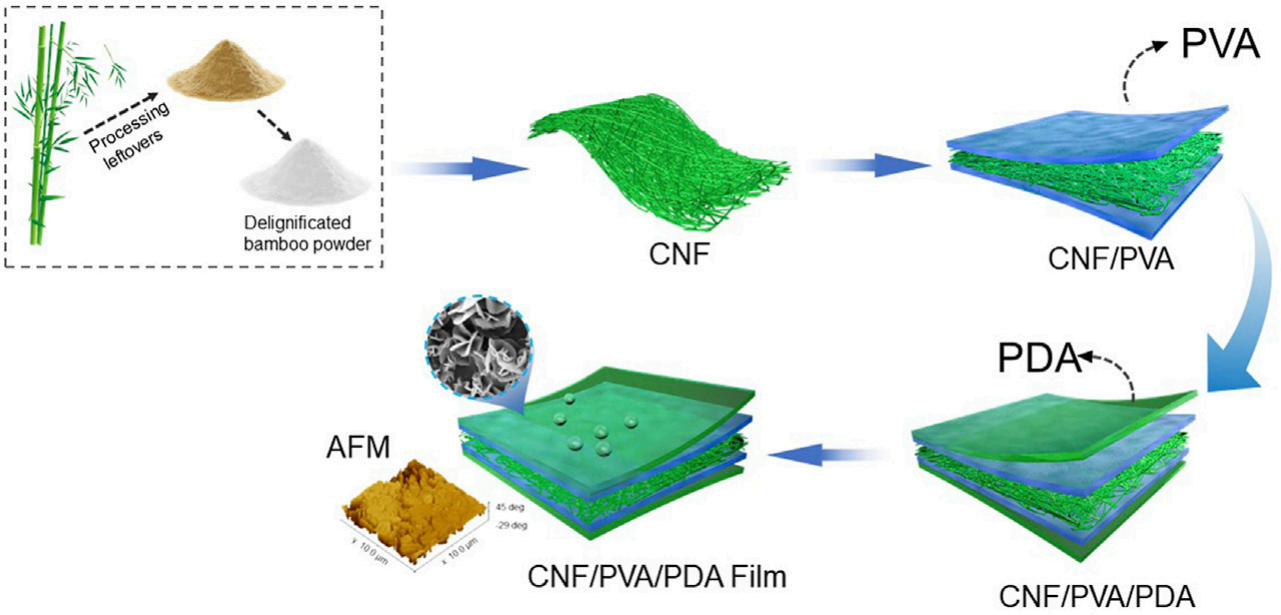
Diagram of the CNF/PVA/PDA preparation process.

Firstly, to remove impurities on the surface of the film, the prepared transparent film was washed and soaked in ethanol for 15 min; Then, 0.2 g dopamine. The diagram of the CNF/PVA/PDA preparation process was shown in Figure 1.

Hydrochloride was added into 10 ml water and stirred evenly; Then 2 g cetyltrimethoxysiloxane and 100 g ethanol were added, then NaOH was added to adjust the pH value to 7.5, and it was mixed thoroughly and stirred evenly. The impregnated film was put into the prepared solution and dipped at room temperature for 48 h. Finally, the film was taken out and dried in the oven at 50°C for 8 min.

### Contact Angle (CA) Test

The contact angle of water on the cross-section of dopamine-modified nanofiber superhydrophobic film was measured by Theta t200 optical contact goniometer (Gothenburg, Sweden, Bailin Technology Co., Ltd.). After drying, the nanofiber superhydrophobic film samples were placed on the loading platform. It will be about 2 µl of deionized water on the surface of the sample. The samples were tested three times in parallel at different positions.

### SEM and Roughness Test

Microscopic electron microscope mainly uses secondary electron signal imaging to observe the surface morphology of the sample. The prepared sample was cut into a film with a width of about 5 mm, a length of 5 mm and a thickness of about 2 mm and fixed on the conductive adhesive where the sample was placed with tweezers. The samples were sprayed with gold for 30 s by vacuum plating equipment. The surface and internal morphology of the samples were observed by Quanta 200 SEM (FEI Company, United States) under 3 KV voltage. The surface structure of the samples was characterized by a roughness meter. The experimental samples are laid on the horizontal platform, and the samples are pressed and fixed with iron blocks. The probe of roughness meter is placed on the surface of the film, and the sampling range is set at 2.5 mm. Ten points of each sample are tested, and the average value of the test results is calculated.

### Diaphaneity Test

UV1900 series UV-Vis spectrophotometer (Shanghai Youke Instrument Co., Ltd.) was used. The wavelength was set at 250–800 nm. The sample was placed in front of the input port of the incident beam, and then the spectrum was derived from another port. Finally, the transmittance value was obtained.

### Fourier Transforms Infrared Spectroscopy

Fourier transform infrared spectroscopy (FTIR) is an effective method to study hydrogen bonding and material compatibility. Use a vertex 80V infrared spectrometer (Brooke, Karlsruhe, Germany) and with the wavenumber range of 500–4,000 cm^−1^.

### Mechanical Tensile Strength Test

The mechanical properties of nano cellulose film before and after modification were tested and calculated. The mechanical properties of the modified nano cellulose films were measured by a computer-controlled electronic universal testing machine (Shimadzu ags-x). The upper and lower clamps of the testing machine first fix and then clamp the sample. At this time, no additional load was set (to reduce the experimental error), then the lower clamp was fixed, and the upper clamp stretched the sample upward until fracture, and the upward stretching speed was set at 200 mm/min.

The following formula is used in the mechanical test:

σ=F/S (1)

S=b∗h (2)

In this formula, *σ* is tensile strength; F is the maximum force on the specimen when it breaks; *S* is the original cross-sectional area of the specimen in the tensile direction; *b* is the initial width of the tensile section, *h* is the initial thickness of the tensile section.

### Self-Cleaning Test

The self-cleaning test method of nano cellulose superhydrophobic film sample is as follows: First, a layer of pencil dust was cut on the surface of the sample as a pollutant, and then a dropper was used to drop small water drops on the surface of the sample to observe whether the water drops can take away the pollutant and the change of the cleanliness of the surface of the sample.

## Results and Discussion

### Contact Angle (CA)Test

**FIGURE 2:**
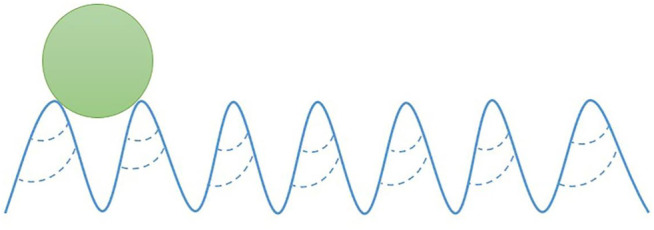
The mechanism of droplet formation on CNF/PVA/PDA film surface.

The results showed that the contact angles of the water on the films of CNF, CNF/PVA and CNF/PVA/PDA (Table 1) were 45.6, 66.3, and 156°, respectively. When droplets dropped on two adjacent nanofibers, the droplets will move along the fibers and spread to other adjacent fibers due to the capillary force generated by the nanofiber boundaries, forming liquid columns. Therefore, the wettability of CNF film surface was controlled by nanofibers, resulting in hydrophilicity. With the decrease of the number of nanofibers, the wetting length of droplets decreased and the contact angle of CNF/PVA film surface increased (Li et al., 2021). Compared with the original CNF film, the hydrophobicity of CNF/PVA/PDA film was improved, and the long-chain alkyl adhered to the surface of the base film under the action of PDA self polymerization complex (Yang et al., 2012), which reduced the surface energy of the film. The wettability of the film surface depended on the geometric potential. When the radius of the protruding surface of the film surface became smaller, a greater force will be generated. When the water droplets approached the nano protrusions on the film surface, the geometric potential will produce a high attraction to attract the water droplets to the film surface. Water droplets can exist between two tiny protrusions. Because the surface energy of CNF/PVA/PDA film was low, water droplets became spherical on the film surface to reduce energy, so as to obtain the liquid air interface. The droplet only contacted the top of the protrusion on the surface of CNF/PVA/PDA film and intercepted the air below into the groove. In addition, the surface roughness can change the surface energy by reducing the surface energy (Figure 2). Therefore, water droplets showed a higher contact angle on the rough surface of CNF/PVA/PDA film.Therefore, the nano cellulose film modified by PDA had superhydrophobic properties.

**TABLE 1:** The contact angle on the surface of CNF, CNF/PVA and CNF/PVA/PDA films.

Name of film	Contact angle of water on the film surface (°)	Image of contact angle
CNF	45.6	Fx1
CNF/PVA	66.3	Fx2
CNF/PVA/PDA	156.0	Fx3

### SEM and Roughness Test

**FIGURE 3:**
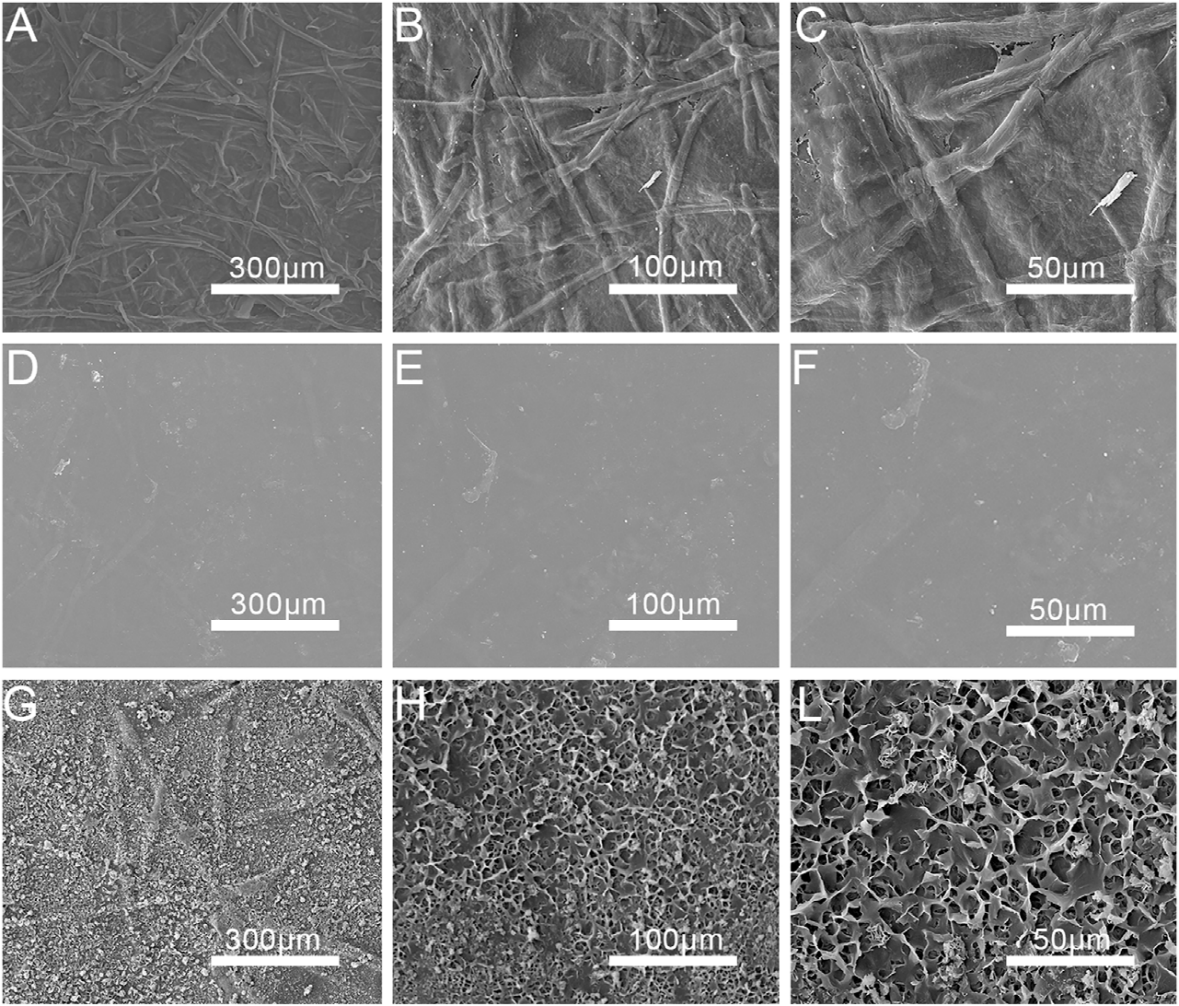
SEM images of CNF, CNF/PVA and CNF/PVA/PDA films **(A)** at 200 magnification; **(B)** at 400 magnification; **(C)** at 800 magnification), CNF/PVA **(D)** at 200 magnification; **(E)** at 400 magnification; **(F)** at 800 magnification) and CNF/PVA/PDA **(G)** at 200 magnification; **(H)** at 400 magnification; **(L)** at 800 magnification) films.

**FIGURE 4:**
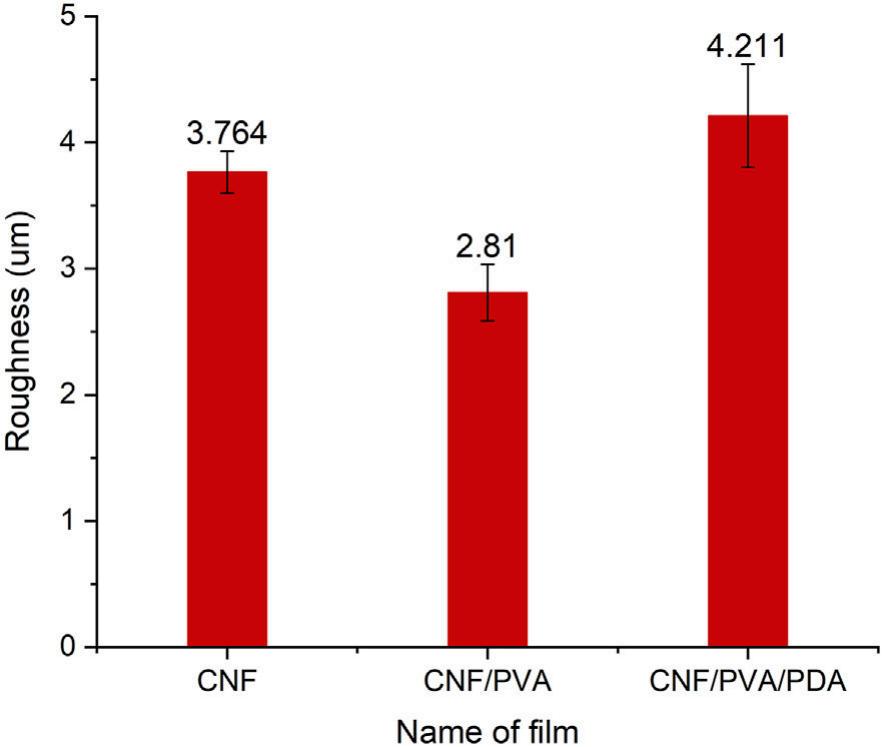
Roughness of CNF, CNF/PVA and CNF/PVA/PDA films.

The surface microstructures of CNF, CNF/PVA and CNF/PVA/PDA films were shown in Figure 3. From Figures 3A–C, the nanofibers on the surface of CNF film are arranged and randomly distributed, thus forming a dense network interwoven structure under 300 um. As can be seen from Figures 3D–F, the surface of the CNF film became more smooth after being soaked in PVA solution, and the internal pores were filled with PVA solution, which became more compact (Mendoza et al., 2021). From Figures 3G,H,L, it could be seen that the surface of the film after silane modification became rough and has small protruding antennae, which was the main reason for the film superhydrophobicity. PVA has good biocompatibility with nano cellulose (Qiu and Netravali, 2012). It can be seen from the data in Figure 4 that the roughness of the modified film obtained by blending PVA solution with nano cellulose film becomes smaller. The alkyls in cetyltrimethoxysilane are bonded to the surface of CNF/PVA film under the action of PDA adhesion, which reduced the surface energy and improves the surface roughness. Therefore, the surface roughness of PDA modified film is improved, the contact between droplets and film surface is reduced, the surface contact angle of film is improved, and the super hydrophobicity of film is realized.

### Diaphaneity Test

As could be seen inFigure 5, the transmittance of CNF film was 34.9% at 800 nm, and then decreased with the decrease of wavelength. It can be seen that CNF films had good UV absorption properties. Filling the gap of nano cellulose transparent paper with polyvinyl alcohol can reduce light scattering and increased the interaction between nano cellulose. At 800 nm, the transmittance of the film reached 44.4%, which improved the transparency of CNF film (Xu et al., 2021). Therefore, PVA modified film can be widely used in the field of flexible transparent materials. After the film was immersed in dopamine solution, the transmittance of the film decreased to 1.5% at 800 nm. This was because the self polymerized complex in dopamine solution deposited on the surface of the film, resulted in the decrease of the transparency of the film.

**FIGURE 5:**
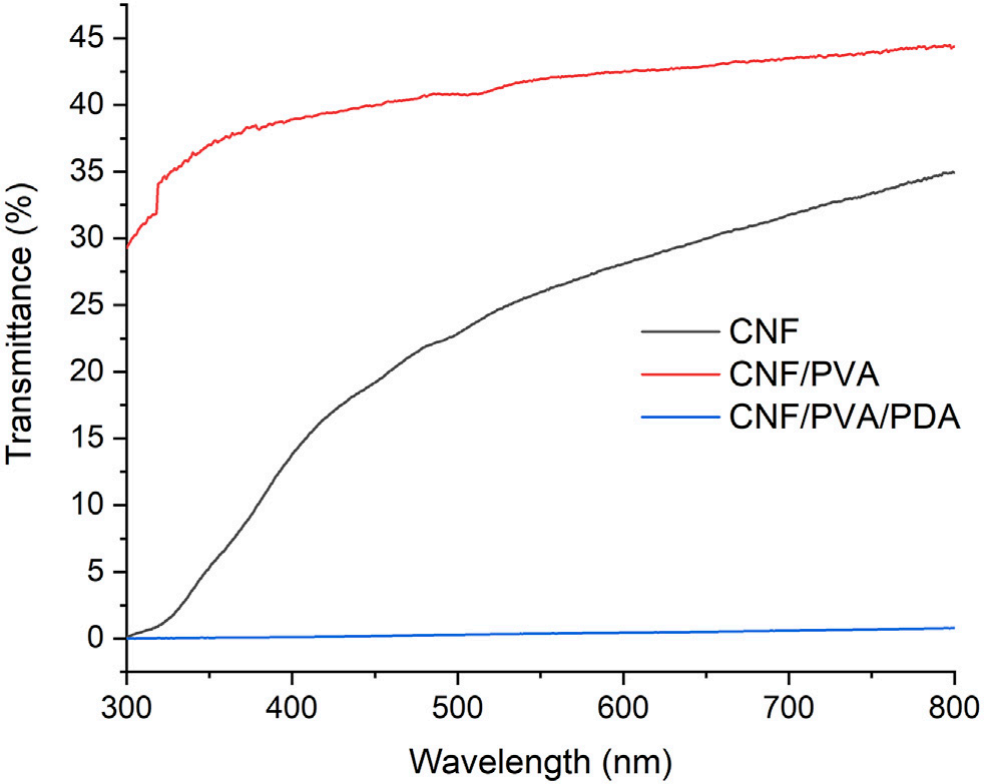
Light transmittances of CNF, CNF/PVA, and CNF/PVA/PDA.

### Fourier Transforms Infrared Spectroscopy

**FIGURE 6:**
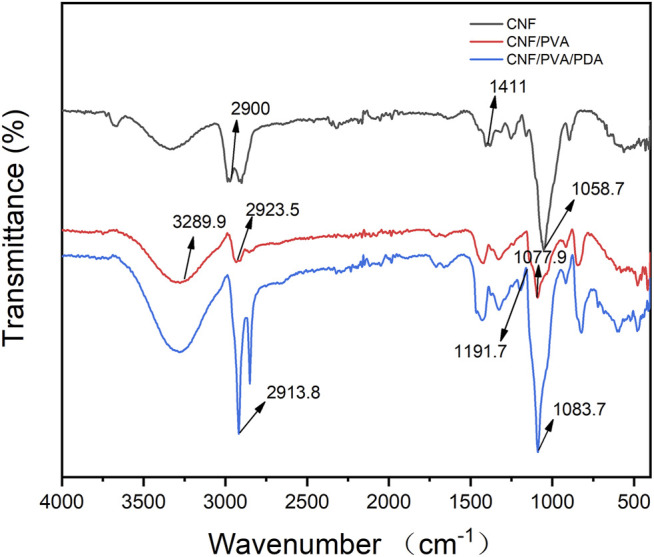
FTIR of CNF, CNF/PVA, CNF/PVA/PDA films.

Figure 6 showed the FTIR spectra of CNF, CNF/PVA, and CNF/PVA/PDA films. It could be seen from the figure that the infrared characteristic peaks of CNF/PVA film were O-H stretching vibration at 3,289.9 cm^−1^, -CH stretching vibration at 2,923.5 cm^−1^, and -C-O stretching vibration at 1,077.9 cm^−1^ (Ibrahim et al., 2013). A 2,900 cm^−1^ in CNF film corresponded to the C-H peak in methylene (-CH_2_) in cellulose, 1,411 cm^−1^ corresponded to the shear vibration of -CH_2_ group, 1,058.7 cm^−1^ was the expansion vibration peak of ether bond C-O-C in cellulose group (Liu et al., 2019b). When the CNF film was immersed in PVA solution, the range of stretching vibration peaks of -OH became wider and moved to the direction of the low wave, which indicated that there was hydrogen bonding force between cellulose and PVA, and they had good biocompatibility. 1,191.7 cm^−1^ corresponded to the C-N deformation tensile vibration peak, the results indicated that the long-chain PDA polymer particles containing catechol group formed by oxidative polymerization of dopamine were grafted onto the fiber of nanocellulosic film, and the polymer was fixed on the film surface by the adhesion of catechol.In the dopamine-modified films, 2,913.8 cm^−1^ corresponded to the corresponding stretching vibration peak of the C-H bond, and the 1,083.7 cm^−1^ region was the asymmetric stretching vibration of the Si–O–Si bond (Medina-Sandoval et al., 2018). The number of alkyl groups on the surface of dopamine-modified films increased significantly, indicating that the surface of modified films has been modified by cetyloxysilane.

### Mechanical Tensile Strength Test

**FIGURE 7:**
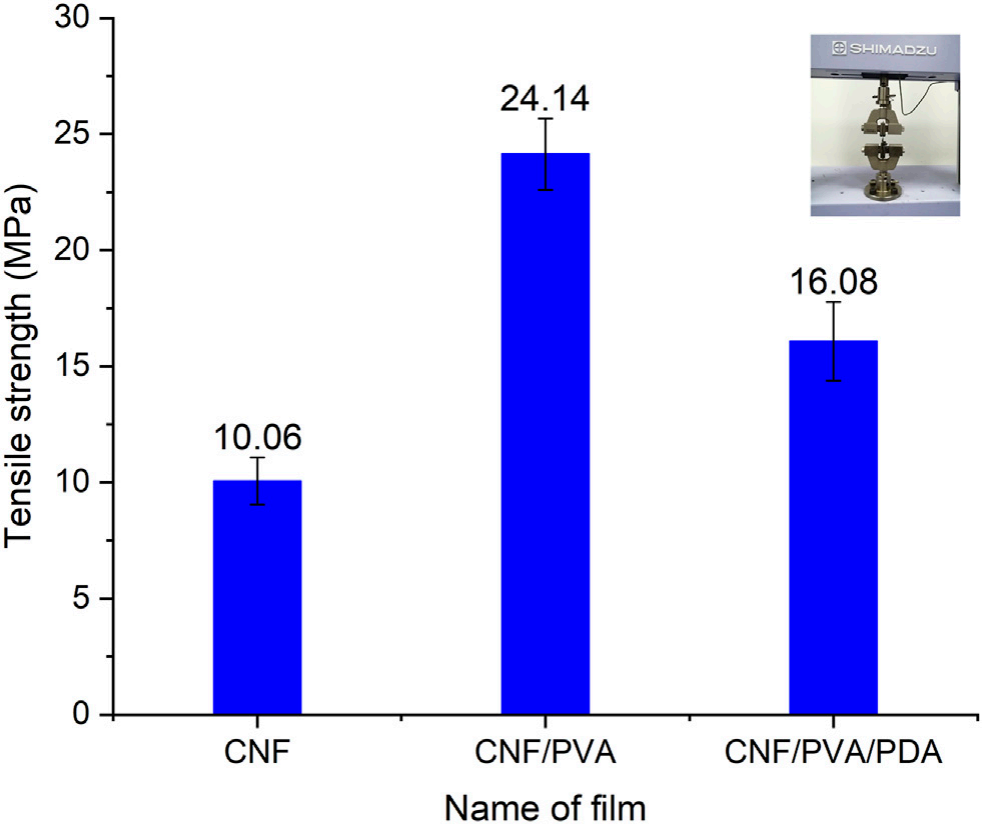
The tensile strength of CNF, CNF/PVA, CNF/PVA/PDA films.

As can be seen in Figure 7, the pull-up strength of CNF/PVA film reached 24.14 MPa, which was significantly higher than that of CNF film. This was because PVA solution can penetrate into nano cellulose to form nano cellulose polymer.There was good interaction between nano cellulose and PVA, and nanopores provided space for the penetration of PVA solution.Meanwhile, the hydroxyl groups in PVA could be filled in the CNF to form hydrogen bonds, the smooth surface formed by the crosslinked CNF/PVA composite film showed that a more stable crosslinked structure was formed, and the interface interaction between different components was significantly improved, thus enhanced the interaction between CNF and PVA, which improved the mechanical properties of the CNF/PVA. Long chain PDA polymer particles containing catechol groups formed by oxidative polymerization of dopamine were grafted onto the fibers of nano cellulose film. These polymers in turn will affect the mechanical properties of the substrate. The polymers were stacked on the film surface, making the film brittle and easier to break. Therefore, the mechanical properties of CNF/PVA/PDA decreased.

### Self-Cleaning Test

**FIGURE 8:**
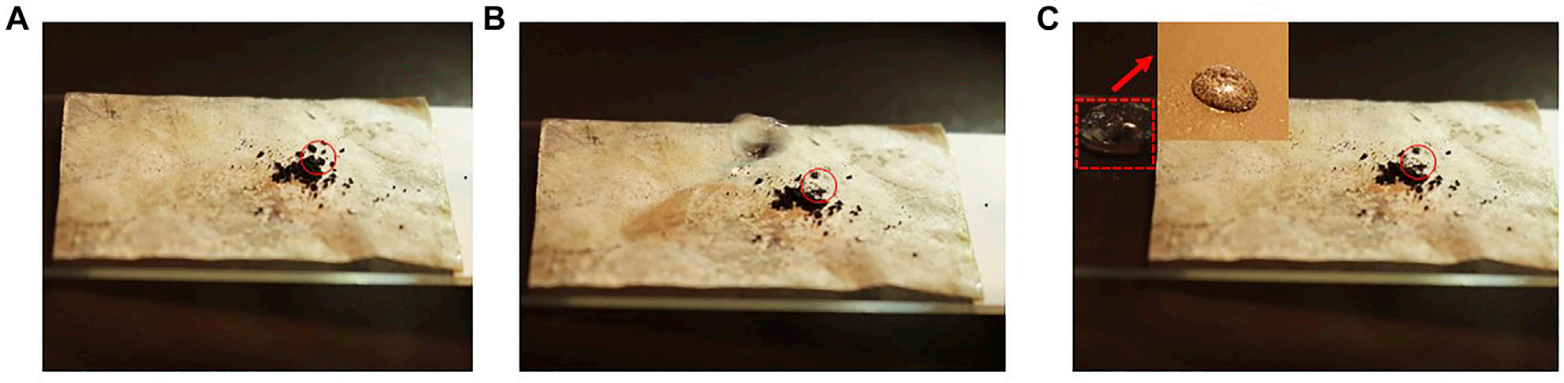
Self-cleaning test CNF/PVA/PDA.

**FIGURE 9:**
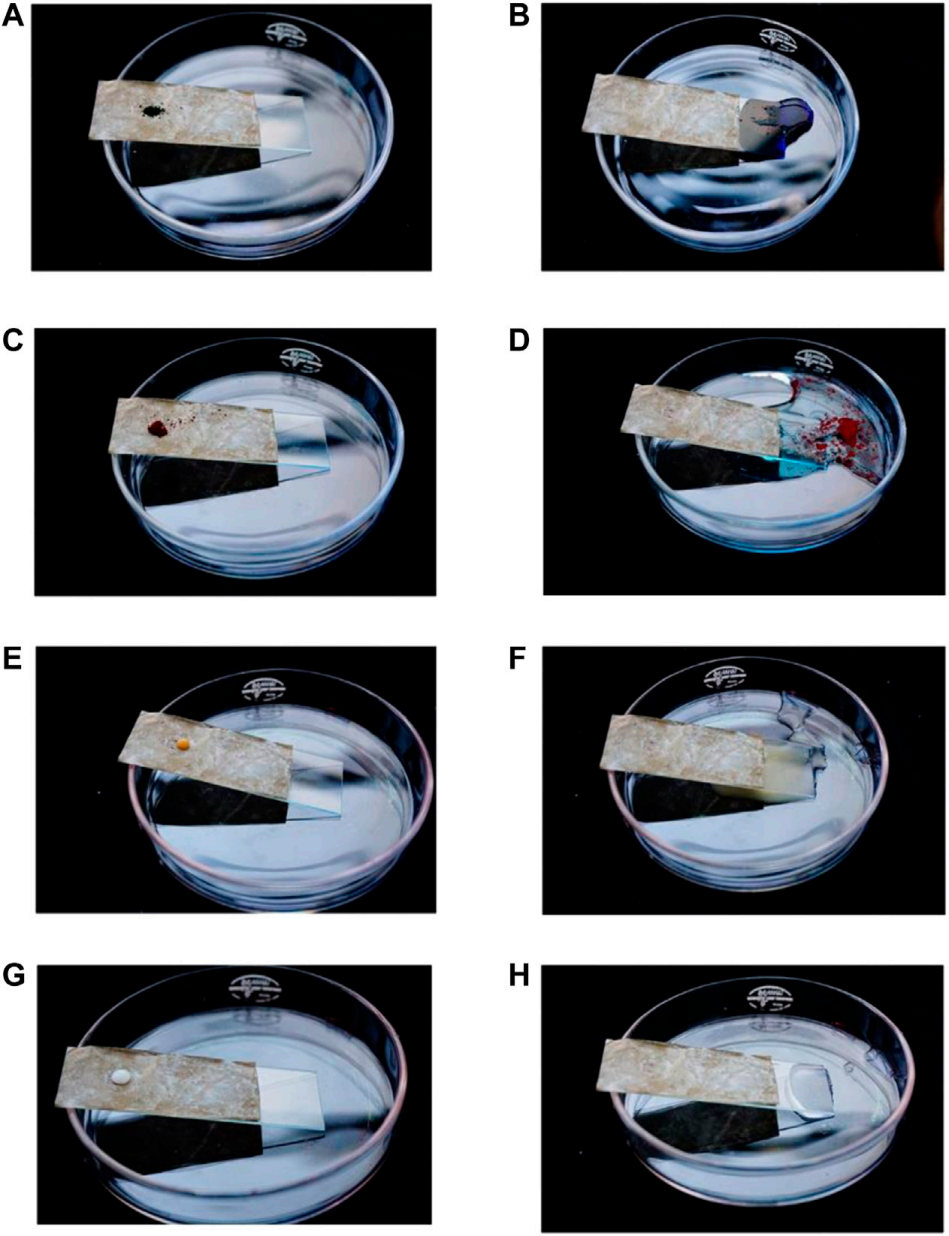
Self-cleaning test CNF/PVA/PDA **(A,B)**: methylene blue; **(C,D)** sudan red; **(E,F)** coffee juice; **(G,H)** milk juice).

One of the excellent properties of the superhydrophobic film was self-cleaning. The self-cleaning test of the PDA modified film is carried out, and the test results are shown in Figure 8.The pencil ash was taken away by the water drops. In the third picture, the pencil ash adheres to the surface of the water drops. It could be seen clearly from the video (Supplementary Video) in the attachment that small water droplets drive the pencil dust on the surface of the film to roll forward together. The long chain alkyl group in the cetyl group was grafted onto the surface of the nanocellulosic film by the surface modification of self-polymerization complex of dopamine, which improved the water resistance and self-cleaning performance of the film surface. Compared with the CNF film, the surface of CNF/PVA/PDA film has higher water contact angle and lower rolling angle. The water drops on the CNF/PVA/PDA film surface will not diffuse freely, keeping the ball-shape, and reduce the contact area with the film surface. When the film surface has a slight inclination, the water bead will drive the pencil ash from the film surface to roll together (see the red square in Figure 8). In order to further verify the self-cleaning performance of the film surface, the author carried out four tests in Figure 9. The pollutants methylene blue, sudan red, coffee juice and milk juice were respectively placed on the surface of a, c, e and g film in Figure 9. The self-cleaning test was carried out according to the same method in Figure 8. It can be found from figures e.g., f and h that water droplets can drive the pollutants on the film surface to roll away together to form a clean surface. These tests fully showed that CNF/PVA/PDA film had excellent superhydrophobic and self-cleaning properties. Therefore, by introducing low surface energy substances on the surface of the cellulose film (PDA), the micro roughness structure was constructed and self-cleaning ability was achieved (Extrand, 2002).

## Conclusion

Bamboo nano cellulose, derived from bamboo, was a renewable and rich biopolymer, which can provide an inexhaustible source for the synthesis of nano fibers. Cellulose was the most popular biopolymer. The filler obtained from cellulose fiber had rich, cost-effective and environmental protection properties, which had great hope for the development of sustainable natural bio based films. In addition, the chemical reagent dopamine hydrochloride selected in this paper was green and environmental friendly, and the preparation cycle was short. The dopamine superhydrophobic film was prepared by one-step method. The method was simple and the preparation conditions are mild. The application of polydopamine with significant hydrophilicity on superhydrophobic surfaces was challenging, but polydopamine had excellent adhesion and can form films on almost all substrate surfaces, which was not affected by the overall size of the substrate. At the same time, polydopamine was used as adhesive to strengthen the adhesion between nanoparticles and film, and a functional film with excellent superhydrophobic and self-cleaning properties was prepared.The nano cellulose film prepared from bamboo fiber was finally superhydrophobic through a series of modification treatments. The results showed the nanofibers on the surface of CNF film were arranged and randomly distributed, thus forming a dense network interwoven structure. In PDA hydrophobic modification solution, an HDTMS was hydrolyzed to a hexadecyl silanol to obtain the polar terminal hydroxyl of Hexadecyl silanol molecule. The -OCH3 terminal group of HDTMS reacted with hydroxyl/H_2_O to form a silanol (Si-OH) bond and further condensed to form a Si-O- Si network. In addition, due to the hydrophilicity of the surface of the nano cellulose film, a large amount of—OH was adsorbed on the surface of the nano cellulose film, resulted in the chemical connection between cetyl groups, thus realized the grafting of cetyl long-chain alkyl groups onto the fibers of the nano cellulose film (Seth et al., 2020). The CNF/PVA/PDA film had good superhydrophobicity and mechanical properties, which indicated that the film can be used in the field of oil-water separation and food packaging in the industry.

## Data Availability

The original contributions presented in the study are included in the article/Supplementary Material, further inquiries can be directed to the corresponding author.
